# Outdoor host seeking behaviour of Anopheles gambiae mosquitoes following initiation of malaria vector control on Bioko Island, Equatorial Guinea

**DOI:** 10.1186/1475-2875-10-184

**Published:** 2011-07-07

**Authors:** Michael R Reddy, Hans J Overgaard, Simon Abaga, Vamsi P Reddy, Adalgisa Caccone, Anthony E Kiszewski, Michel A Slotman

**Affiliations:** 1 Department of Epidemiology and Public Health, Yale University, New Haven, CT USA; 2 Medical Care Development International Inc., Malabo, Equatorial Guinea; 3 Department of Mathematical Sciences and Technology, Norwegian Life Sciences University, Ås, Norway; 4 National Malaria Control Programme, Ministry of Health and Social Welfare, Malabo, Equatorial Guinea; 5 Department of Entomology, Texas A&M University, College Station, TX USA; 6 Department of Ecology and Evolutionary Biology, Yale University, New Haven, CT USA; 7 Department of Natural and Applied Sciences, Bentley University, Waltham, MA USA

## Abstract

**Background:**

Indoor-based anti-vector interventions remain the preferred means of reducing risk of malaria transmission in malaria endemic areas around the world. Despite demonstrated success in reducing human-mosquito interactions, these methods are effective solely against endophilic vectors. It may be that outdoor locations serve as an important venue of host seeking by *Anopheles gambiae *sensu lato (s.l.) mosquitoes where indoor vector suppression measures are employed. This paper describes the host seeking activity of anopheline mosquito vectors in the Punta Europa region of Bioko Island, Equatorial Guinea. In this area, *An. gambiae *sensu stricto (s.s.) is the primary malaria vector. The goal of the paper is to evaluate the importance of *An gambiae *s.l. outdoor host seeking behaviour and discuss its implications for anti-vector interventions.

**Methods:**

The venue and temporal characteristics of host seeking by anopheline vectors in a hyperendemic setting was evaluated using human landing collections conducted inside and outside homes in three villages during both the wet and dry seasons in 2007 and 2008. Additionally, five bi-monthly human landing collections were conducted throughout 2009. Collections were segregated hourly to provide a time distribution of host-seeking behaviour.

**Results:**

Surprisingly high levels of outdoor biting by *An. gambiae *senso stricto and *An. melas *vectors were observed throughout the night, including during the early evening and morning hours when human hosts are often outdoors. As reported previously, *An. gambiae *s.s. is the primary malaria vector in the Punta Europa region, where it seeks hosts outdoors at least as much as it does indoors. Further, approximately 40% of *An. gambiae *s.l. are feeding at times when people are often outdoors, where they are not protected by IRS or LLINs. Repeated sampling over two consecutive dry-wet season cycles indicates that this result is independent of seasonality.

**Conclusions:**

*An. gambiae *s.l. mosquitoes currently seek hosts in outdoor venues as much as indoors in the Punta Europa region of Bioko Island. This contrasts with an earlier pre-intervention observation of exclusive endophagy of *An. gambiae *in this region. In light of this finding, it is proposed that the long term indoor application of insecticides may have resulted in an adaptive shift toward outdoor host seeking in *An. gambiae *s.s. on Bioko Island.

## Background

In 2004, Marathon Oil Corporation in conjunction with its industrial partners and the Government of Equatorial Guinea embarked upon the Bioko Island Malaria Control Project (BIMCP), a public-private partnership designed to reduce the burden of malaria on the population of Bioko Island [1]. The BIMCP is comprised of a combination of vector suppression and disease reduction strategies, and various operational research components, e.g. on insecticide resistance mechanisms. A comprehensive monitoring and evaluation system is in place, also including the entomological components of malaria transmission, which play an integral role in assessing the epidemiological impact of the various intervention activities.

Anti-vector interventions were initially focused on indoor residual spraying (IRS) of pyrethroids in nearly 80% of all houses on Bioko Island [1,2]. Homes were sprayed once annually with Deltamethrin™ (Bayer Crop Science Inc., Isando South Africa) or Fendona™ (alpha-cypermethrin; BASF South Africa PTY Ltd.) [3,4]. In 2005, a high frequency of genetic "knockdown" resistance (*kdr*) was documented among *Anopheles gambiae *s.s. mosquito vectors, prompting a switch to twice yearly spraying of Ficam™ (bendiocarb; Bayer Crop Science Inc. Isando South Africa), a carbamate insecticide [2,3]. Spraying occurred between February through July and August through December, the highest transmission periods of the year [4]. An island-wide distribution of deltamethrin-treated long-lasting insecticidal nets (LLINs) was started in late 2007 and completed the following year. Data regarding IRS spray coverage and LLIN usage is collected as part of the BIMCP's ongoing monitoring and evaluation activities. These include an annual parasitemia survey among < 15 year old children and household demographic questionnaires. Surveys include inspection of homes for the presence/absence and condition of LLINs. Such surveys have been carried out on an annual basis at roughly the same time of the year since the inception of the BIMCP in 2004 [2]. A high daily usage rate in excess of 75% was initially observed, however this quickly diminished after one year with about one third of respondents reporting they had slept under a treated bed net the previous evening (Kleinschmidt, unpublished). In addition to anti-vector interventions, improved case detection and management, and the distribution of free anti-malarial drugs comprise an important component of the overall anti-malaria campaign.

These anti-vector interventions and disease reduction strategies have substantially reduced childhood mortality on Bioko Island since initiation of the BIMCP [1,2]. Despite marked decreases in prevalence, malaria remains endemic and presently represents the most significant threat to human health [1,2,4]. Transmission of *Plasmodium falciparum *on Bioko Island continues to occur year-round and is considered intense and stable. Entomological monitoring indicates that *An. gambiae *s.s. serves as the primary vector on the majority of the island, with *Anopheles melas *the dominant vector in several coastal zones [4], (Slotman, unpublished). The frequency of *Anopheles funestus *declined following the start of the intervention and is currently very low [4], (Slotman, unpublished).

Limited reports indicate that *An. gambiae *s.s., which is widely regarded as a primarily endophagic and endophilic vector, historically exhibited a high degree of indoor feeding and resting on Bioko Island [5,6]. Nonetheless, blood fed *An. gambiae *have been collected exiting houses in window traps, indicating a degree of exophily [5]. These reports, as well as pre-intervention entomological monitoring data, provided the rationale for anti-vector interventions that include an active IRS programme and supplemental distribution of LLINs. Indoor residual insecticides and barrier methods have been proven effective against endophilic vectors such as *An. gambiae *s.s., *An. arabiensis*, and *An. funestus *[7-12]. Where exophagy represents a substantial proportion of feeding behaviors, particularly at times when people are active and outdoors, intra-domiciliary interventions are often not effective in substantially reducing malaria transmission [11].

*Anopheles gambiae *s.s. have been documented to enter homes in the early evening hours, tending to feed in the late evening hours, and exit in the early morning hours [9-12]. Studies comparing *An. gambiae *indoor *vs *outdoor biting behaviour in various countries report between 18% and 100% endophagy [5,13-15]. On Bioko Island however, no outdoor biting was detected in a previous study using human bait collections [5]. As a contrasting example, Wanji *et al *found that only 29% to 35% of *An. gambiae *bite indoors in Cameroon [14], with no difference detected between the dry and wet season. On the other hand, data from Ghana suggest that *An. gambiae *is more endophagic in the dry season than in the wet season [15]. Thus, although *An. gambiae *is typically considered an endophagic mosquito, this trait appears to vary between locations, seasons, or both.

In many locations, *An. gambiae *s.s. exhibits an extremely high degree of anthropophagy [16,17]. Despite this distinct host preference, *An. gambiae *will feed on cows and dogs in the absence of available human hosts, [18-20]. Such plasticity may also apply to resting behaviours and IRS and LLIN interventions may induce changes in endophilic tendencies. Some insecticides have excito-repellent properties, that may induce selection for outdoor biting behaviours, where indoor-based insecticidal measures selectively kill indoor feeding/resting mosquitoes [11,12]. It may therefore be that outdoor locations could become an important venue for host-seeking *An. gambiae *s.l. where indoor anti-vector measures are widely employed. Accordingly, the host-seeking activity of anopheline mosquito vectors was evaluated in three villages in the area of Punta Europa, Bioko Island; an area of the island that has sustained high levels of transmission since the initiation of anti-malaria activities, despite major reductions throughout the rest of the island [3]. Indoor *vs *outdoor human landing collections (HLC) were carried out during both the wet and dry seasons in 2007, 2008 and 2009 to determine the venue and temporal characteristics of host-seeking by *An. gambiae *s.l. mosquitoes in the presence of indoor-based anti-vector interventions. These studies were conducted in conjunction with the BIMCP's entomological monitoring efforts and operational research programme on insecticide resistance and mosquito behaviour in response to anti-vector activities. The goal of this study was to assess the importance of outdoor-feeding where indoor anti-vector interventions such as IRS and LLINs are currently in use.

## Methods

**Figure 1:**
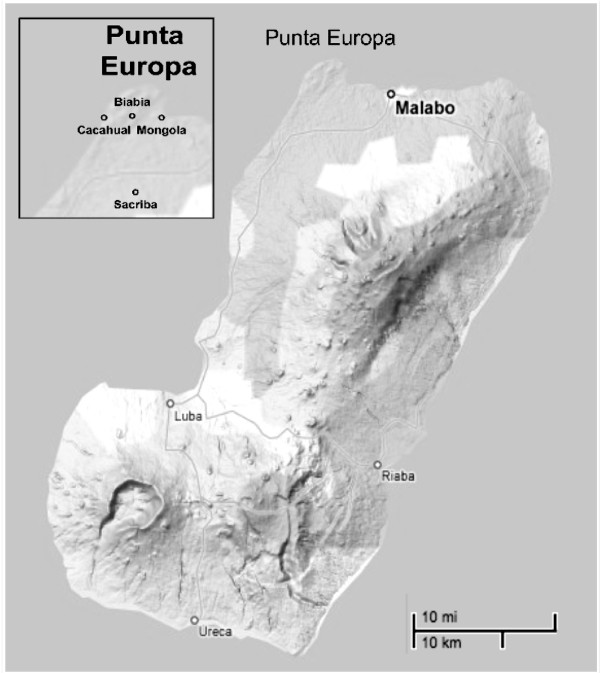
**Map of study villages in Punta Europa, Bioko Island, Equatorial Guinea**. Also indicated is Sacriba, the location of the collections made by Molina et al [5].

Human landing collections were performed in three rural villages in close proximity; Mongola (3°45.88788'N,8°43.30314'E), Cacahual (3°45.84354'N,8°41.12424'E), and Biabia (3°45.69354'N,8°42.32994'E) in the Punta Europa region of Bioko Island (Figure 1). Collections were made during four consecutive nights, both in the early part of the dry season (November 2007) and the end of the rainy season (September 2008). Five pairs of human landing collectors were used in each village per evening during the 2007-08 collections. Each pair collected mosquitoes from residences at least 100 metres away from other collection sites. Indoor collectors were positioned in a central room within the home, often in the sleeping quarters. Outdoor collectors were located several metres outside the perimetre of the same home. Collections were initiated shortly after dusk (19:00 hours) and continued through the early morning hours (06:00 hours), with mosquitoes collected hourly and stored in individual collection tubes. From March to November 2009, indoor and outdoor human landing collections were performed in a similar way as in 2007-2008, but at four houses, every second month, and in Mongola village only. Outdoor collections took place immediately outside the house, often under verandas. The wet season was defined as April-October and the dry season as November-March, based on annual rainfall means from the meteorological station at Malabo International Airport (located in the immediate vicinity of Mongola) during 2000-2007.

Collectors were recruited from each of the communities. Ethical approval for this study was granted by authorities from the National Malaria Control Programme (NMCP) of the Equatoguinean Ministry of Health and Social Welfare. The lead entomologist (SA) of the NMCP was present and provided supervisory support for all collection activities.

Anopheline mosquitoes were identified based on morphology and stored in 80% ethanol prior to transport to the laboratory for molecular analyses. Heads and thoraces were dissected and subjected to DNA extraction using a QIAGEN DNeasy Blood & Tissue Kit by microcentrifuge or on a QIAGEN Biosprint (QIAGEN Sciences Inc., Germantown, MD). A diagnostic PCR followed by restriction enzyme digest was used for species identification within the *An. gambiae *s.l. complex and to determine the molecular form (M/S) of *An. gambiae *s.s. [21,22].

Endophagy, exophagy, and nocturnality (i.e. the proportion of mosquitoes collected between 21:00 and 05:00 hours, were calculated for each collected mosquito species in both 2007/2008 and 2009. Statistical analyses were performed using SPSS software 18.0 release (IBM Corporation, Somers, NY) [23]. Chi-square tests were performed to test if the proportion of mosquitoes collected indoors vs. outdoors was significantly different in 2007-2008 and the 2009 collections. A Z-test of means was used to determine whether the mean number of mosquitoes collected differed significantly during the wet versus the dry season in 2009. A logistic regression was performed to determine whether the proportion of host seeking events occurring indoors and outdoors changed throughout the course of the collection night [24,25].

## Results

### 2007 and 2008 collections

A total of 653 mosquitoes were collected by human landing captures from the three villages in Punta Europa in 2007 and 2008. *An. gambiae *s.s. was the dominant species (n = 587; 89.9% ± 2.3%; 95% CI), with all specimens belonging to the M molecular form. The remainder of the collections was composed entirely of *An. melas *(n = 66; 10.1% ± 2.3%; 95% CI).

**Figure 2:**
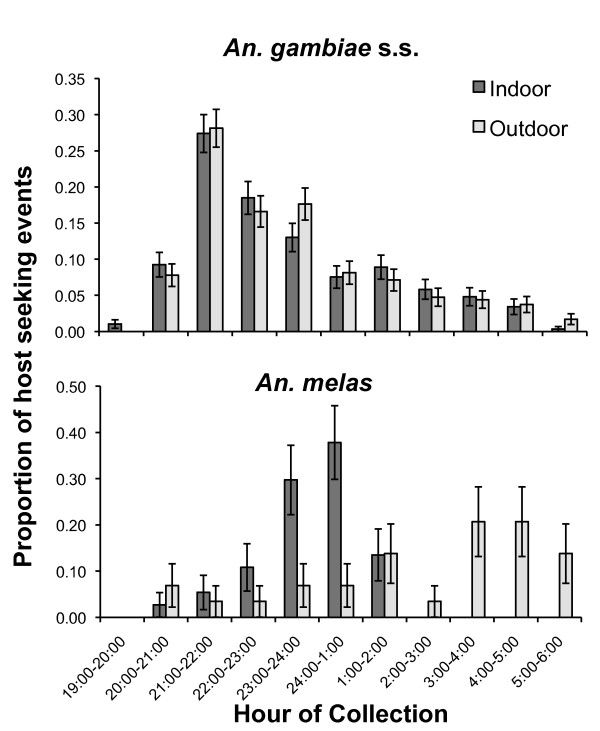
**Time segregated human collections in Punta Europa, 2007 and 2008**. Error bars represent standard error of the proportion.

In Figure 2, the proportion of *An. gambiae *s.s. and *An. melas *collected indoor *vs *outdoor is represented by hour, starting at 19:00 to 06:00 hours. The proportion of *An. gambiae *s.s biting indoors or outdoors was similar throughout the night as indicated by the overlapping standard errors of means. Collections in both indoor and outdoor locations increased rapidly during the early evening with peak biting occurring between 21:00 and 22:00 hours and steadily declining thereafter. Among *An. melas *on the other hand, indoor biting steadily increased through the early evening hours, peaking around midnight and dropping off precipitously thereafter. Outdoor biting among *An. melas *was relatively low and uniform during the early evening and increasing slightly during the early morning hours. A logistic regression was performed to test whether biting rates differed significantly between indoors and outdoors venues throughout the night. No significant difference was detected (p > 0.05) among *An. gambiae *s.s. or *An. melas*. Nocturnality of both *An. gambiae *s.s. and *An. melas *was nearly 90% among both *An. gambiae *s.s. and *An. melas *[25] (Table 1).

**Table 1:** Summary of 2007-2008 and 2009 *An.gambiae *s.l. collections in Punta Europa, Bioko Island

	2007-2008		
***An. gambiae s.s.***	**n**	**Proportion**	**± S.E**.

**Endophagy**	292	0.497	0.029
**Exophagy**	295	0.503	0.029
**Nocturnality**	528	0.899	0.012

***An. melas***			

**Endophagy**	37	0.561	0.082
**Exophagy**	29	0.439	0.092
**Nocturnality**	59	0.894	0.038

	**2009**		

***An. gambiae s.s.***			

**Endophagy**	608	0.504	0.020
**Exophagy**	598	0.496	0.020
**Nocturnality**	1042	0.864	0.010

Nocturnality is defined as the proportion of mosquitoes collected indoors and outdoors between the hours of 21:00 and 5:00 hours [25].

**Figure 3:**
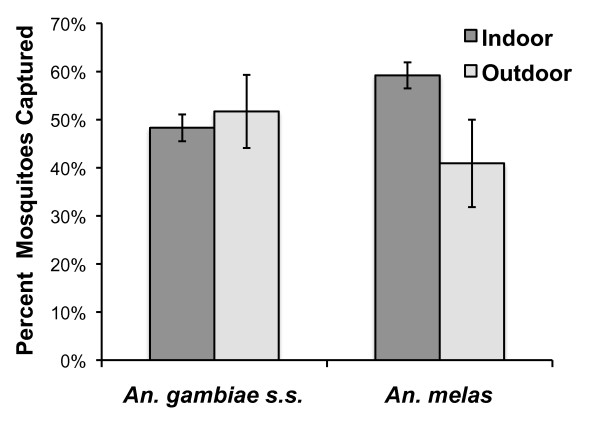
**Proportion of anophelines collected indoors and outdoors in Punta Europa, 2007 and 2008**. Error bars represent standard error of the proportion.

The relative proportion of *An. gambiae *s.s. and *An. melas *mosquitoes collected indoors versus outdoors is presented in Figure 3. *An. gambiae *s.s. host-seeking was nearly equal between indoor (49.7% ± 2.9% SE) and outdoor (50.3% ± 2.9% SE) venues, whereas a greater proportion of *An. melas *mosquitoes were collected inside (56.1% ± 8.2% SE) than outside homes (43.9% ± 9.2% SE) (Table 1). However, the proportions of mosquitoes biting indoors or outdoors between species were not significantly different (χ^2 ^= 0.947; p < 0.330).

### 2009 collections

A total of 7,604 (3,172 indoors *vs *4,432 outdoor) anophelines were collected during March, May, July, September and November of 2009 in the village of Mongola in the Punta Europa region. A sub-sample of the total collection (15.9%; n = 1,206 mosquitoes) was identified to species and molecular form. Similar to the 2007 and 2008 collections, *An. gambiae *s.s. was the dominant species (> 99%), with all *An. gambiae *s.s. belonging to the M molecular form. About 200 mosquitoes out of the 7,604 collected did not belong to the *An. gambiae *s.l. complex and were excluded from the analyses. The nocturnality of both *An. gambiae *s.s. was greater than 86% (Table 1), consistent with similar findings in Tanzania [24].

**Figure 4:**
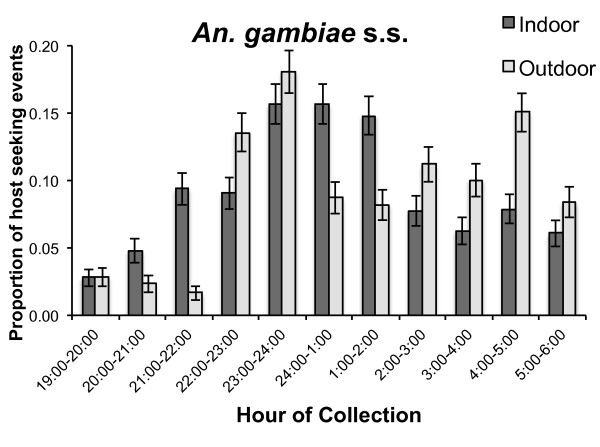
**Time segregated human collections in the village of Mongola, 2009**. Error bars represent standard error of the proportion.

Figure 4 shows the combined 2009 hourly collections for the total number of anopheline mosquitoes analysed (n = 1,206). The proportion of *An.gambiae *s.s. collected indoor *vs *outdoor is represented by hour, starting at 19:00 to 06:00 hours. Indoor biting predominated between 24:00 and 02:00 hours, whereafter outdoor biting was proportionally greater through to the early morning hours. Both indoor and outdoor biting increased rapidly during the early evening with peak biting occurring between 23:00 and 24:00 hours. A logistic regression indicated that the proportion of host-seeking events did not differ significantly between indoors and outdoors venues throughout the night (p > 0.05).

**Figure 5:**
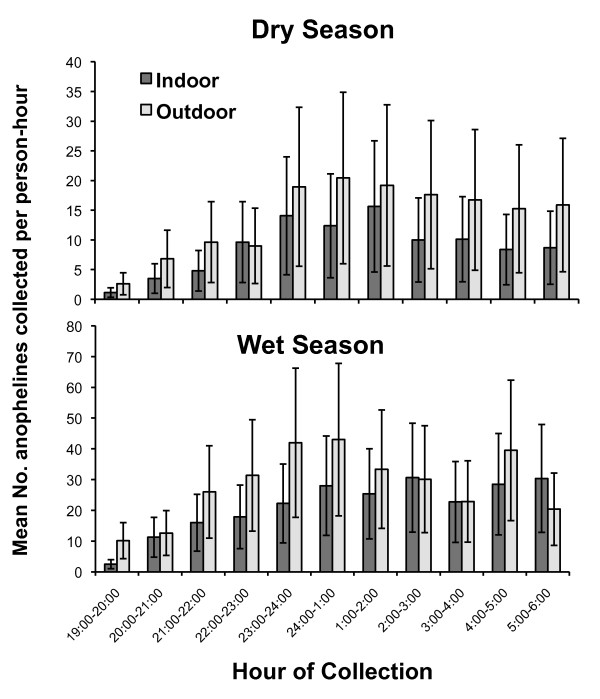
**Hourly human landing collections by season and venue in Mongola, Punta Europa, Bioko Island, 2009**. Error bars represent the standard error of the mean.

Hourly collections were performed in both the wet and dry seasons, which are presented in Figure 5. Significantly more mosquitoes were collected during the wet (n = 4375) than the dry season (n = 3229); χ^2 ^= 7.7; df = 1 p < 0.006). The proportion of anophelines collected outdoors was significantly higher during the wet season (56.2% ± 1.5%; 95% CI) than the dry season (43.8% ± 1.5%; 95% CI) (Z = 11.653; p < 0.001). Combined wet and dry season collections showed that between 19:00 and 24:00 hrs, 38.5% (± 3.9%; 95% CI) and 41.6% (± 3.9%; 95% CI) of *An. gambiae *s.s. were collected outdoors and indoors, respectively.

**Figure 6:**
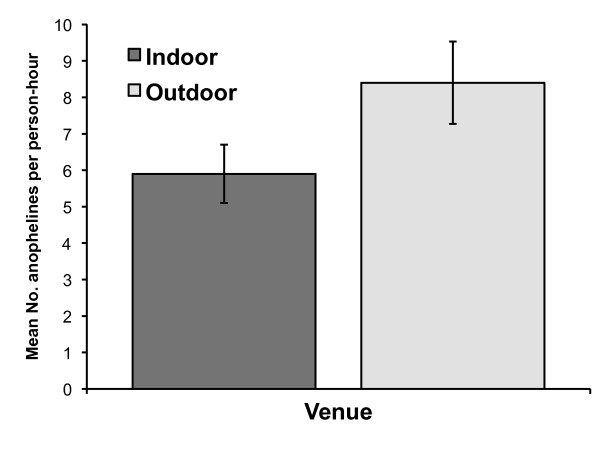
**Mean number of anophelines collected indoors and outdoors in Mongola, Punta Europa, Bioko Island, 2009**. Error bars represent the standard error of the mean.

A larger number of host seeking mosquitoes were collected outdoors than indoors in 2009, as shown in Figure 6. Overall, the mean number of mosquitoes per person-hour collected outdoors (8.4 anophelines per person-hour ± 1.1 SE) was significantly higher than for indoor collections (5.9 anophelines per person-hour ± 0.8 SE; p < 0.024).

## Discussion

This study indicates that *An. gambiae *and *An. melas *readily seek hosts in outdoor venues in the Punta Europa region of Bioko Island. The relevance of outdoor biting behaviour of nocturnal mosquitoes to vector suppression depends greatly on whether outdoor biting coincides with human outdoor activity. Previous studies have reported that host seeking activity of *An. gambiae *peaks around midnight [5,6], which corresponds with the results from the 2009 collection. However, in this study nearly 40% of all host-seeking mosquitoes were collected outdoors between the early evening and midnight.

Entomological monitoring on Bioko Island and continental Equatorial Guinea has yielded important, albeit anecdotal insight into human behaviour. No data on the amount of outdoor human activity in relation to exposure to mosquitoes were collected in conjunction with this study so the degree of human-mosquito contact at times when people are not protected by indoor anti-vector interventions is difficult to quantify. However, human activity outside the home into the late evening hours is very common in Punta Europa and throughout Equatorial Guinea. Future studies of human behaviour would provide important insights into human activity and other potential risk factors associated with outdoor biting.

Many domiciles also serve as outdoor, semi-enclosed lounge areas where adults and children congregate into the late evening hours. This was observed in all three communities where collections were performed in 2007, 2008 and 2009. Outdoor activity was particularly apparent in the study village of Mongola, where a large number of outdoor eating and drinking establishments are present and frequented by local villagers and expatriate workers. Collections did not extend into the hours between 06:00 to 19:00 hrs and it is also possible some feeding activity extends into the early daylight hours, when humans are also active outdoors. This extensive outdoor human activity when *An. gambiae *biting is at its peak means that a substantial amount of feeding by anopheline vectors is taking place outdoors, where indoor-based interventions are not effective. Therefore, the ability of the current interventions to reduce malaria transmission is hampered by the observed outdoor biting behaviour.

*An. gambiae *mosquitoes are typically considered to exhibit highly endophagic and endophilic behaviours and to feed primarily on human hosts [9,26], even though endophagy has been shown to be a highly variable trait [13-15,19,24,25]. High levels of exophagy and opportunistic feeding on animals have been observed in *An. gambiae *s.l. mosquitoes in specific ecological contexts throughout sub-Saharan Africa [14,18,19,25]. These studies examined the potential impact of behavioural resistance on intervention efficacy, but did not attempt to demonstrate a causal effect of concerted indoor-based anti-vector interventions, such as IRS or LLINs on selection for insecticidal avoidance mechanisms. In order to assess such a relationship both pre-intervention and post-intervention analyses of biting behaviours are required.

Although this study did not include pre-intervention data, Molina *et al *[5] conducted an admittedly limited study comparing indoor *vs *outdoor biting of anopheline vectors a decade before the start of the BIMCP intervention in the village of Sacriba. This village is located within eight kilometres from the Punta Europa study sites (Figure 1). Despite collecting indoor biting mosquitoes, Molina *et al. *did not collect any outdoor biting anophelines, Other authors annecdotally reported also that no anophelines were observed feeding outdoors on Bioko Island in 1998-1999 [6].

In early 2004, 48.6% of *An. gambiae *s.l. in the Punta Europa area belonged to the M molecular form, 50.2% belonged to the S molecular form and 1% was *An. melas *(Slotman, unpublished). No S-form *An. gambiae *s.s. were observed in the current collections, meaning that only the M form of *An. gambiae *remains, at least in Mongola. This confirms previous observations that S-form populations declined following the initiation of anti-vector measures [2]. Further, given that almost 50% of Punta Europa samples consisted of the M molecular form in 2004, the marked difference in outdoor feeding between the current study and previous observations cannot be explained by differences in abundance of the two molecular forms of *An. gambiae *historically and in the present [4,5].

These results raise the question whether *An. gambiae *s.s. actively seek hosts in outdoor venues in response to ongoing, indoor vector suppression efforts. Although no genetic polymorphism for endo/exophagy has been identified to date [11,20,27], a continuum of behavioural tendencies have been observed among anopheline populations [4,13-15,18,24]. Indoor application of insecticides could result in selection for increased exophagy and/or changes in the biting time of *An. gambiae *s.l. [28-30]. Such behaviours may be the result of effective IRS and/or LLIN interventions that predominantly kill mosquitoes that feed or rest indoors, resulting in a reproductive advantage for those mosquitoes that opportunistically feed outdoors. The efficacy of indoor anti-vector measures may in part, explain the large proportion of outdoor host seeking observed in this study.

Historical precedence for taxon-level replacement due to anti-vector activities exists in Africa and Asia. Predominantly endophagic, anthropophagic vector populations have been dramatically altered by IRS and LLIN interventions so that the residual population consists largely of more exophagic, zoophagic sibling species. These residual populations are less affected by intra-domiciliary insecticide-based interventions. *An. funestus *has been replaced by *An. rivulorum *and/or *An. parensis *following the introduction of indoor residual spraying of insecticides on at least three distinct occasions in South Africa, Kenya and Tanzania [9,31-33]. More recently, long-term use of LLINs in Tanzania and Kenya has resulted in the near disappearance of *An. gambiae*, leaving almost pure populations of *An. arabiensis *[27,34,35]. In another recent example from the Pacific, *An. punctulatus *and *An. koliensis *were eliminated by past IRS campaigns, leaving only *An. farauti*, which is now exhibiting a modified behaviour and very weak susceptibility to IRS and LLIN interventions [36].

It is well known that insect populations have the capacity to adapt rapidly to the use of insecticides. Insecticidal interventions have the capacity to exert significant selection pressure for genetic resistance when intensively applied [3,11,37,38]. One example is the recent rapid increase in frequency of target site resistance to pyrethroids and DDT insecticides on Bioko Island [3,37,38]. It is conceivable that concurrent selection for behavioural resistance resulting in exophagic tendencies has occurred among *An. gambiae *s.s. not killed upon contact with indoor residual insecticides. Alternatively, the observed patterns in host-seeking behaviour could represent a response to the excito-repellent effect (i.e. contact irritancy and spatial repellency) of residual insecticides, diverting otherwise endophagic mosquitoes to seek hosts outdoors [39]. Excito-repellency is well documented for DDT and pyrethroids [40-43]. However, bendiocarb, the carbamate used in IRS activities on Bioko Island since 2005, does not have an excito-repellent effect on *An. gambiae *and *An. pseudopunctipennis *[44,45]. Recent results also indicate that *An. gambiae *s.l. on Bioko Island remain susceptible to bendiocarb as measured by WHO-standardized bioassays (Overgaard, unpublished). Further, G119S, the genetic polymorphism conferring acetylcholinesterase (*ace-1*) target site insensitivity to carbamates was notably absent among anopheline mosquitoes collected in 2009 (Slotman, unpublished).

In light of this evidence it is likely that long-term indoor application of insecticides on Bioko Island has resulted in a shift to outdoor host seeking among residual *An. gambiae *s.s populations due to selection pressure imposed by the toxicity of bendiocarb used in the IRS campaign conducted by the BIMCP. The IRS campaign achieved high coverage in the area and was conducted twice a year, whereas the LLIN distribution resulted in low bed net use.

The extensive outdoor host seeking observed in this study may in part explain the high parasitemia rates in the Punta Europa area, despite intensive vector suppression efforts [3]. Such results have led the BIMCP to evaluate a number of potential additional interventions to reduce malaria transmission in outdoor venues. These include deployment of insecticide-treated wall hangings in outdoor bars, personal repellents and source reduction.

## Conclusions

Indoor-based residual insecticide and bed net-based approaches have been proven effective against epidemiologically important, endophilic anopheline vectors. However, where blood feeding and resting occurs outdoors in significant proportions, indoor-based interventions do not suffice to reduce malaria transmission to desired levels [3]. The data presented in this study, along with the recent work by Russell *et al. *2011 [24] suggest that the long-term indoor application of residual insecticides contributes toward an increased tendency for outdoor feeding among malaria vector populations. This is expected to erode the efficacy of indoor-based interventions over time, much as increased insecticide resistance would [29]. Regardless of whether *An. gambiae *on Bioko Island experienced a shift in host seeking behaviour, or was already partially exophagic on Bioko Island prior to the start of intervention activities, the outdoor biting behaviour documented here indicates that it is imperative to explore possibilities for outdoor anti-vector interventions, in combination with ongoing IRS and LLIN distribution. This is currently being done by the BIMCP and should be recommended for other anti-malarial programmes as well.

## Competing interests

The authors declare that they have no competing interests.

## Authors' contributions

MRR: Conceived and planned the study, performed the field collections and molecular analyses of the 2007 & 2008 collections, and prepared the first draft. HJO: Participated in the study design, performed the 2009 field collections, provided editorial input, and helped draft the manuscript. SA: Participated in the study design and supervised field collections 2007-2009 and provided editorial input. VPR: Performed the molecular analyses of the 2009 field collections. AC: Supervised the molecular analyses of the 2007 & 2008 collections and contributed to manuscript preparation. AEK: Participated in the study design, statistical analysis assistance, and provided editorial input. MS: Supervised and planned the study, supervised the molecular analysis of the 2009 field collections and contributed to manuscript preparation. All authors read and approved the final manuscript.
